# The power of a touch: Regular touchscreen training but not its termination affects hormones and behavior in mice

**DOI:** 10.3389/fnbeh.2023.1112780

**Published:** 2023-03-16

**Authors:** Sophia Marie Quante, Viktoria Siewert, Rupert Palme, Sylvia Kaiser, Norbert Sachser, S. Helene Richter

**Affiliations:** ^1^Department of Behavioural Biology, University of Münster, Münster, Germany; ^2^Department of Biomedical Sciences, University of Veterinary Medicine, Vienna, Austria

**Keywords:** cognitive enrichment, anxiety-like behavior, glucocorticoids, anticipation, enrichment loss, stress inoculation, negative contrast, touchscreen technology

## Abstract

Touchscreen-based procedures are increasingly used in experimental animal research. They not only represent a promising approach for translational research, but have also been highlighted as a powerful tool to reduce potential experimenter effects in animal studies. However, to prepare the animals for a touchscreen-based test, an often time-consuming training phase is required that has itself been shown to cause increased adrenocortical activity and anxiety-like behavior in mice. While these findings point at a potentially negative effect of touchscreen training at first glance, results have also been discussed in light of an enriching effect of touchscreen training. The aim of the present study was therefore to shed more light on recently reported touchscreen training effects, with a particular focus on the termination of the training routine. Specifically, we investigated whether the termination of regular touchscreen training could constitute a loss of enrichment for mice. Thus, we assessed fecal corticosterone metabolites (FCMs), exploratory-, anxiety-like and home cage behavior in touchscreen-trained mice in comparison to food restricted and *ad libitum* fed mice, as a restricted diet is an integral part of the training process. Furthermore, we compared these parameters between mice that were continuously trained and mice whose training was terminated 2 weeks earlier. Our results confirm previous findings showing that a mild food restriction increases the animals' exploratory behavior and shifts their activity rhythm. Moreover, touchscreen training was found to increase FCM levels and anxiety-like behavior of the mice. However, no effect of the termination of touchscreen training could be detected, a finding which contradicts the enrichment loss hypothesis. Therefore, we discuss two alternative explanations for the findings. Yet, the current state of knowledge is not sufficient to draw final conclusions at this stage. In compliance with the refinement endeavors for laboratory animals, further research should assess the severity of touchscreen procedures to ensure a responsible and well-founded use of animals for experimental purposes.

## 1. Introduction

Touchscreen-based procedures are increasingly used in animal research (Bussey et al., 2008). Due to the similarities to human testing techniques [e.g., CANTAB (Fray et al., 1996)], they hold a high translation potential, with some tasks being already successfully translated to rodents (Armbruster et al., 2012; Richter et al., 2014). Besides this, touchscreen procedures have been highlighted as a powerful tool to reduce potential experimenter effects in animal studies, thereby representing an important refinement strategy (Richter et al., 2014). However, in order to prepare the animals for a touchscreen-based test, an often time-consuming and intense training phase is required that consists of several weeks of daily training (Richter et al., 2014). As it has already been shown that such routinely applied and predictable procedures can have extensive effects on the animals (Bassett and Buchanan-Smith, 2007), it cannot be excluded that the touchscreen training itself can also affect the experimental outcome. Indeed, there are two studies that already report an influence of regular touchscreen training on hormones and behavior in mice. Both show effects on hypothalamus-pituitary-adrenal (HPA) axis activity of touchscreen trained mice across the day, which was highest during the anticipation of a training session (Mallien et al., 2016; Krakenberg et al., 2021). Moreover, we reported increased anxiety-like behavior in touchscreen trained mice in a previous study (Krakenberg et al., 2021). At first glance, these findings might point at a detrimental effect of touchscreen training, indicating impaired welfare in these animals (Paul et al., 2005). However, in light of the so-called “stress inoculation” hypothesis, the findings regarding HPA axis activity could also indicate the opposite effect, namely an enriching effect of touchscreen training. More precisely, according to this hypothesis, mild daily stress is assumed to lead to a higher coping ability with environmental stressors, thus contributing to improved welfare (Crofton et al., 2015; Mallien et al., 2016). In line with these thoughts, we previously developed an alternative explanation, suggesting that an “enrichment loss effect” could account for the increased anxiety-like behavior in the touchscreen trained mice. More specifically, the tests to assess anxiety-like behavior were conducted with a temporal distance of 2 weeks to the termination of the touchscreen training. If the mice indeed perceived touchscreen training as enriching, a termination of training would pose a loss of enrichment, which might be reflected in increased anxiety-like behavior (Krakenberg et al., 2021). This explanation seems to be especially reasonable, as touchscreen training represents a cognitive challenge and cognitively active animals are assumed to be of greater risk to suffer from enrichment removal (Nicol, 1996).

Following up our previous study (Krakenberg et al., 2021), we here aimed to investigate the effects of touchscreen training termination. Hence, we not only included touchscreen trained and control mice in our experiment, but also compared continuously touchscreen trained mice with mice whose training was terminated 2 weeks earlier. As in the mentioned study, we conducted a battery of standardized tests concerning anxiety-like and exploratory behavior and determined fecal corticosterone metabolites (FCMs), which reflect adrenocortical activity (Palme, 2019). However, besides analyzing home cage activity we extended our focus to include stereotypies, which can be used as an indicator for impaired welfare (Latham and Mason, 2010). In line with the stated literature, we hypothesized touchscreen trained mice to display differences in behavioral, as well as endocrinological measurements compared to control mice. Furthermore, we hypothesized continuously touchscreen trained mice to differ from mice whose training was previously terminated concerning the mentioned parameters.

## 2. Animals, materials and methods

### 2.1. Animals and housing conditions

The study included 72 male C57BL/6J mice, ordered from Charles River Laboratories (Research Models Services, Germany GmbH, Sulzfeld, Germany) at postnatal day (PND) 28. Mice were delivered in 3 batches, i.e., at three different time points, with always 24 mice per batch. From then on, all individuals were housed singly. Although male mouse housing is a topic of controversial discussion in research (Kappel et al., 2017; Melotti et al., 2019), single housing was chosen in this study, as the applied mild food restriction holds the potential to increase aggressive interactions. The 3 animals that initially shared the same cage were treated as matched triplets for the following experimental phase. The cages (Makrolon Typ III cages: 38 × 22 × 15 cm^3^) contained wood shavings as bedding material (TierWohl Super, J. Rettenmaier and Söhne GmbH & Co KG, Rosenberg, Germany), a paper tissue, a wooden stick, and a semi-transparent red plastic house (Mouse HouseTM, Tecniplast Deutschland GmbH, Hohenspeißenberg, Germany). In addition, a transparent red plastic tunnel (Mouse Tunnel Red, Plexx B.V., Elst, Netherlands) was added to the cages 1 week before the experimental phase started. Water and food (Altromin 1324, Altromin Spezialfutter GmbH & Co. KG, Lage, Germany) were offered *ad libitum*, except during specific phases of the experiment that required a restricted feeding regime (for details see below). The housing room was maintained at a reversed dark/light cycle with lights off at 9 a.m., a temperature of ~22°C, and a relative humidity of about 50%.

### 2.2. Experimental design

**Figure 1:**
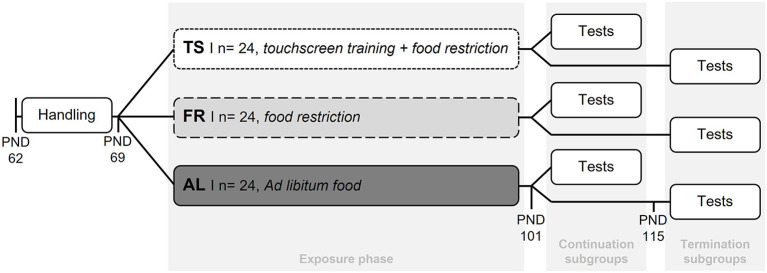
Experimental design. After the mice were habituated to tunnel handling (PND 62–69) they were assigned to one of three groups. TS, touchscreen trained and mildly food restricted (90–95% of ad libitum feeding weights) group; FR, food restricted group (90–95% of *ad libitum* feeding weights) without touchscreen training; AL, *ad libitum* fed group without touchscreen training. After the 5-week long exposure phase, half of the mice from each group were tested in behavior tests, while their feeding routines and touchscreen training remained unaffected. These subgroups were termed “continuation subgroups”. For the remaining mice from the TS group the touchscreen training was terminated at this point (PND 101). These mice, as well as the remaining mice from the AL and FR group, were tested in behavior tests 2 weeks later (start: PND 115) and termed “termination subgroups”.

Following the experimental design of Krakenberg et al. (2021), all mice were habituated to tunnel handling 1 week before the start of the different feeding routines and the touchscreen training. This was done by gently guiding the mouse into the tunnel that was already located in the home cage for enrichment purposes. Tunnel handling was found to be less stressful compared to the commonly used tail handling technique (c.f. Hurst and West, 2010). For the subsequent exposure phase (start: PND 69), mice were assigned to one of 3 groups: a touchscreen trained group (TS, *n* = 24), a food restricted control group (FR, *n* = 24), or an *ad libitum* fed control group (AL, *n* = 24) (Figure 1). TS mice were mildly food restricted to 90–95% of their *ad libitum* body weights and trained in 5 sessions per week, each with a duration of 15 min. A restricted diet is usually applied during touchscreen training to increase the animals' motivation to gain food rewards (Horner et al., 2013). Although any touchscreen paradigm could have been used to investigate the effects of the regular training, the present study exemplarily used a Cognitive Judgement Bias task, which is originally used to assess decision making under ambiguity (Krakenberg et al., 2019a). For a detailed description of the touchscreen task please see Supplementary material. The two control groups (FR and AL) were included to differentiate between effects from the touchscreen training and the mild food restriction. Both never received touchscreen training sessions. As TS mice, FR mice were restricted to 90–95% of their *ad libitum* body weights (for details see Supplementary material), while AL mice were fed an *ad libitum* diet. After the exposure phase (PND 101), half of the mice from each group were tested in a battery of standardized behavior tests concerning anxiety-like and exploratory behavior, while their feeding routines and touchscreen training continued as before. These subgroups were termed “continuation subgroups”. The remaining half of mice from each group were labeled “termination subgroups”. Here, the touchscreen training was terminated for mice from the TS subgroup (PND 101) and only the mild food restriction continued. The feeding routines of FR and AL mice from the termination subgroups remained unaffected. To investigate the effects of touchscreen training termination, the termination subgroups were tested for their anxiety-like and exploratory behavior with a temporal distance of 2 weeks to the point of touchscreen training termination (PND 115).

#### 2.2.1. Fecal corticosterone metabolites (FCMs)

To study the effects of touchscreen training on adrenocortical activity, the animals' FCMs were monitored non-invasively over the course of the experiment. Similar to Krakenberg et al. (2021), “baseline” and “reaction” FCMs were measured. The expected effects of touchscreen training and the feeding regime, respectively, can be assumed to subside within only 90 min (Mallien et al., 2016). Therefore, the here obtained “baseline” FCMs reflect corticosterone levels ~2 h after training and/or the respective feeding routine had been conducted. “Reaction” FCMs represent corticosterone levels directly before (anticipation value), during, and after the respective experimental procedures. As during the dark phase, a peak of FCM concentrations in response to an event can be found 4–6 h later (Touma et al., 2003), feces collection was adjusted accordingly. Before the start of the exposure phase, FCM “baseline” values were determined for all animals (PND 62). In order to investigate the effect of touchscreen training on adrenocortical activity, FCM “baseline” and “reaction” values were measured in the middle of the exposure phase (baseline: PND 84; reaction: PND 87). These two measurements were repeated after the exposure phase (baseline: PND 104; reaction: PND 106), before behavioral testing started for the continuation subgroups.

##### 2.2.1.1. Fecal sampling

To collect the feces, regular Makrolon Typ III cages, filled with a small amount of bedding, were prepared. A new mouse house, wooden stick and paper tissue were placed inside each cage. After the mouse was transferred to the cage with the help of the tunnel from the home cage, this tunnel was also left in the sampling cage as enrichment. Before the mouse was handled, it was checked that no old droppings were attached to the tunnel. For food restricted mice, food leftovers were transferred to the sampling cage and back to the home cage later, if still present. Water was offered *ad libitum*. The sampling cages were closed with the lid from the home cage, stacked inside the home cage and placed back to the mouse's rack position. After exactly 3 h, the mice were transferred back to their home cages, together with the enrichment from the sampling cage. Subsequently, the fecal boli were collected with gloves, whereby all feces from one sampling cage were stored in a distinct, labeled 1.5 ml Eppendorf tube (Eppendorf AG, Hamburg, Germany) at −20°C.

##### 2.2.1.2. Extraction and analysis of fecal corticosterone metabolites

For the analysis of the FCMs, the wet weight of the fecal samples was determined (scale: 510-23, Kern, Ballingen, Germany; weighing capacity: 300 g, resolution: 0.001 g). Subsequently, the samples were dried for 2 h at 80°C in an oven (Modell 500, D-06061, Memmert, Schwabach, Germany). The dried feces were weighed again and stored in 2.0 ml safe-lock Eppendorf tubes. In the following, the feces were pulverized with a bead mill (TissueLyser LT, Qiagen, Hilden, Germany) by using a stainless steel ball (diameter: 7 mm, Qiagen, Hilden, Germany). 50 mg of the feces powder was then filled into a new 1.5 ml Eppendorf tube and mixed with 1 ml methanol (80%). If there was < 50 mg of powdered feces available in a sample, the amount of methanol was adjusted. The mixture was vortexed for 30 min (Multi-vortex, V-32, Kisker, Steinfurt, Germany) and centrifuged for 10 min with a speed of 5,200 rpm (Centrifuge 5415 R, Eppendorf, Hamburg, Germany). Subsequently, 500 μl of the supernatant that contained FCMs were transferred to a 2.0 ml safe-lock Eppendorf tube and stored at −20°C. In the following, FCM concentrations were analyzed by using a 5α-pregnane-3β,11β,21-triol-20-one enzyme immunoassay (see Touma et al., 2003, 2004).

#### 2.2.2. Home cage behavior

To examine the animals' activity rhythm and the occurrence of stereotypies in relation to touchscreen training and its termination, home cage behavior recordings were taken before, during and after the exposure phase (PND 64-66, 99-101 and 114-115). Please note that the last recording time only included the mice from the termination subgroups, as mice from the continuation subgroups were already tested in the behavior tests. The home cages of the mice were filmed for 24 h and the videos were analyzed concerning activity and stereotypies by using *instantaneous scan sampling* with intervals of 30 min (Bateson and Martin, 2021). Data from the time between 9 and 11 a.m. was not assessed, due to the feeding routines and the touchscreen training being performed. During the analysis of the home cage behavior the experimenter was blinded regarding mice from the FR and TS group. As the experimenter could see continuously filled feeding racks in the cages of AL mice on the videos, AL mice were identifiable on the recordings. On the videos, a mouse was considered *active*, when it showed any kind of motion, excluding tiny whisker, ear or tail movements (Feige-Diller et al., 2020). A stereotypy was counted when a mouse showed *circling, route tracing, jumping* or *back flipping* (Table 1).

**Table 1:** Ethogram for home cage behavior.

**Activity**	
Active	The mouse shows any kind of locomotor activity (e.g., climbing, gnawing, grooming, digging, etc.). In addition, if a mouse is covered, movements of the nesting material, food pellets or rising bubbles in the water bottle are also considered as active behavior.
Inactive	The mouse does not show any kind of locomotor activity. Tiny whisker, ear or tail movements are excluded.
**Stereotypies**	
Circling	The mouse shows circular locomotion and completes more than one full circle three times in a row. The mouse can perform this behavior while climbing on the cage lid or while showing locomotion on the floor.
Route tracing	The mouse moves along the same path that it did before at least three times.
Jumping	The mouse pushes itself upwards with its hind legs, followed by a phase when the whole body of the mouse does not touch the ground (the tail can still touch the ground) at least three times in a row.
Back flipping	The mouse throws its head and body backwards and completes a full round in the air before landing on its paws and repeats this sequence at least three times.

Given are the behaviors and their definitions that were used for the home cage analysis.

#### 2.2.3. Behavioral tests

In the behavioral test phase, the mice's anxiety-like and exploratory behavior was tested in the Elevated plus maze test (EPM; continuation subgroups: PND 108, termination subgroups: PND 122), Open field test (OF; continuation subgroups: PND 111, termination subgroups: PND 125), and Free exploration test (FET; continuation subgroups: PND 112/113, termination subgroups: PND 126/127). All behavior tests were performed between 2 p.m. and 4 p.m. in a separate test room. The order in which the mice were tested was always randomized. For the transport to the test room, a Makrolon Typ II cage (floor space: 23 × 17 × 14 cm), covered with a black blanket to protect the mice from the light in the hallway, was used. Before the start of each test, the mouse spent 1 min inside the transportation cage for acclimatization, to make sure that all animals were in the same state of arousal when being tested (Izídio et al., 2005). Inside the test room, the behavior of the mice was recorded and tracked by a camera (DMK 22AUC03, The Imaging Source, Bremen, Germany) and a tracking software (ANY-maze Video Tracking Software, version 6.32, Stoelting Co., Wood Dale, United States), so that the experimenter could leave the room. Before the first mouse, as well as between all mice, the apparatus was cleaned with 70% ethanol and paper tissues.

##### 2.2.3.1. Elevated plus maze test (EPM)

The apparatus of the EPM (Pellow et al., 1985; Lister, 1987) was plus-shaped and made out of gray plastic, with two opposing closed arms (35 × 6 cm), two opposing open arms (35 × 6 cm) and a square center zone (6 × 6 cm). The closed arms were surrounded by 15 cm high walls and the open arms by a 0.2 cm high border, to secure the mice when leaning over the edge. The whole apparatus was elevated 60 cm above the ground and placed in a fixed orientation inside a white plated wooden arena (80 × 80 × 40 cm), to ensure that fallen mice could not escape. The test apparatus was illuminated from above with a light intensity of ~28 Lux. The mouse was put in the center zone of the test apparatus, facing the closed arm pointing away from the experimenter. The test duration was 5 min. The relative time spent on the open arms, the relative number of entries into the open arms and the distance traveled on the open arms were taken as measures of the animals' anxiety-like behavior. The sum of entries made into the open and closed arms of the apparatus and the total distance traveled was taken as a measure of their exploratory locomotion (Rodgers and Johnson, 1995). Parameters regarding the center of the EPM were excluded from the analysis, due to their ambiguous possibilities of interpretation (Shepherd et al., 1994).

##### 2.2.3.2. Open field test (OF)

The apparatus of the OF (Archer, 1973; Treit and Fundytus, 1988) was square-shaped, with a floor space of 80 × 80 cm, a wall height of 40 cm and made out of gray plastic. The space 20 cm from the walls was defined as the peripheral zone and the space in the middle of the arena (40 × 40 cm) was defined as the center zone. The test arena was illuminated from above with a light intensity of ~30 Lux. The mouse was placed inside the front left corner of the arena, facing the wall. The test duration was 5 min. The time spent in and the numbers of entries made to the center of the apparatus were taken as measures of the animals' anxiety-like behavior. The total distance traveled was taken as a measure of their exploratory locomotion (Krakenberg et al., 2019b).

##### 2.2.3.3. Free exploration test (FET)

Similar to the OF, the apparatus of the FET (Griebel et al., 1993) was square-shaped, with a floor space of 60 × 60 cm and a wall height of 34 cm. The arena was made of white plated wood and had a hole on the right rear corner, where a square-shaped transparent plastic tunnel (10 × 15 × 9 cm) was connected. To this tunnel, the home cages of the tested mice could be connected. Therefore, the mice were put into special cages with a slider during the last cage change before the test. Inside the arena, the space 15 cm from the walls was defined as the peripheral zone and the space in the middle of the arena (30 × 30 cm) was defined as the center zone. The test arena was illuminated from above with a light intensity of ~35 Lux. While the mouse spent 1 min inside the transportation cage, the home cage was connected to the test apparatus. Then the mouse was put back into its home cage and the tracking was started. The test had a duration of 15 min. The time spent in and the latency the enter the arena were taken as measures of the animals' anxiety-like behavior. The total distance traveled and the numbers of entries made to the arena were taken as measures of their exploratory locomotion (Krakenberg et al., 2019b).

### 2.3. Statistics

For the statistical analysis, heteroscedasticity and normal distribution of residuals were examined descriptively and with the Shapiro-Wilk normality test. If the assumptions for parametric analyses were not met, data was transformed. One parameter could not be transformed (FET: arena entries) but simulation studies showed mixed-effect models to be relatively robust against violations of distributional assumptions (Knief and Forstmeier, 2018; Schielzeth et al., 2020). Therefore, the analysis of behavior tests and hormone data was conducted using linear mixed-effect models (LMM). Data concerning the home cage behavior of the animals was analyzed descriptively.

FCM sample points during the handling and exposure phase were analyzed with “group” (3 levels: AL, FR, TS) as fixed factor and “batch” as random factor. Batch refers to the number of animals that were supplied by the animal breeder on the same date (3 levels: 1st, 2nd, 3rd delivery). Afterwards the Tukey's test was performed for *post hoc* comparisons.


$$\begin{array}{l} FCMs\;\sim \;group+\left(1|batch\right) \end{array}$$

For the two FCM sample points after the exposure phase, where the animals were split into subgroups, as well as for the behavior test data, the analysis was conducted with “group” and “subgroups” (2 levels: continuation, termination) as fixed factors and “batch” as random factor, followed by Tukey's test for *post hoc* comparisons.


$$\begin{array}{l} FCMs\;\sim \;group*subgroups+\left(1|batch\right)\\ Behaviour\;\sim \;group*subgroups+\left(1|batch\right) \end{array}$$

Degrees of freedom were always rounded to the nearest integer and differences were considered significant for *p* ≤ 0.05. Significance levels of 0.05 < *p* ≤ 0.1 were considered a trend. To provide a standardized measure for the reported effects, partial eta squared (η^2^_*p*_) was calculated (Lakens, 2013). Analyses were carried out using the statistical software R [version 3.5.0 (R Core Team)] and R studio [version 2021.09.0 + 351 (R Core Team)]. The used sample size was determined by performing a power analysis (G^*^Power Version 3.1.9.6; Faul et al., 2009). We aimed to detect large effects (*f* = 0.4) with a power of 80% regarding the interaction (group^*^subgroups), which requires a sample size of 11 individuals per group. The presented study included 12 mice per group, to account for possible exclusions during the touchscreen training.

## 3. Results

### 3.1. Touchscreen training influenced FCMs

**Figure 2:**
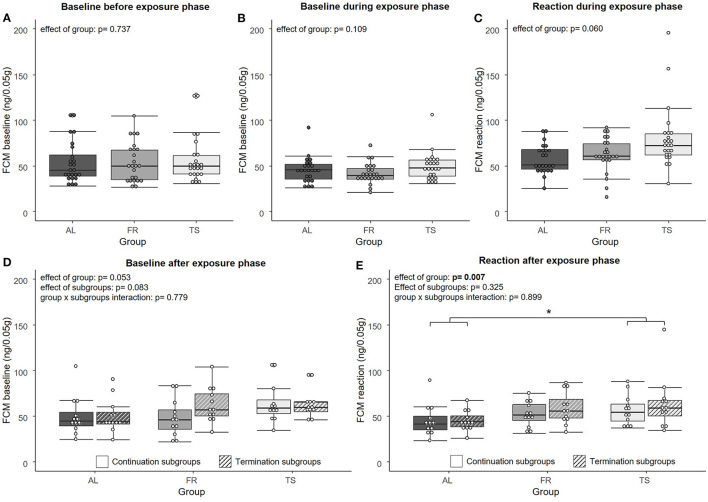
Fecal corticosterone metabolites (FCMs). **(A)** FCM “baseline” values before the exposure phase, **(B)** FCM “baseline” values during the exposure phase, **(C)** FCM “reaction” values during the exposure phase, **(D)** FCM “baseline” values after the exposure phase, **(E)** FCM “reaction” values after the exposure phase. AL, *ad libitum* fed mice; FR, food restricted mice; TS, touchscreen trained mice. Sample sizes: *n* = 24/group for **(A–C)** and *n* = 12/group for **(D, E)**. Box plots show the median (lines in boxes), the 25 and 75% quartiles (boxes), the minima and maxima (whiskers) and individual data points (circles). Statistics: LMM. *p ≤ 0.05.

Regarding the FCM analysis, no significant effect of group was found on FCM “baseline” concentrations [LMM, *F*_(2,67)_ = 0.307, η^2^_*p*_ = 0.009, *p* = 0.737] before (Figure 2A) and during the exposure phase [LMM, *F*_(2,69)_ = 2.294, η^2^_*p*_ = 0.062, *p* = 0.109] (Figure 2B). For the FCM “reaction” values from the exposure phase, a trend for an effect of group was detected [LMM, *F*_(2,66)_ = 2.936, η^2^_*p*_ = 0.082, *p* = 0.060] (Figure 2C). On a descriptive level, FR mice showed slightly increased levels compared to AL mice and TS mice showed the highest levels of all three groups. After the exposure phase, FCM “baseline” values revealed a trend for an effect of group [LMM, *F*_(2,63)_ = 3.078, η^2^_*p*_ = 0.089, *p* = 0.053] and subgroups [LMM, *F*_(1,63)_ = 3.099, η^2^_*p*_ = 0.047, *p* = 0.083], but no effect of group × subgroups interaction [LMM, *F*_(2,63)_ = 0.251, η^2^_*p*_ = 0.008, *p* = 0.779] (Figure 2D). In general, TS mice tended to have higher values than mice from the other two groups and mice from the termination subgroups tended to have higher values than mice from the continuation subgroups. The FCM “reaction” values from the sample point after the exposure phase showed a significant effect of group [LMM, *F*_(2,66)_ = 5.334, η^2^_*p*_ = 0.139, *p* = 0.007], with TS mice having significantly higher values compared to AL mice (*p* = 0.007) and FR mice showing a trend for higher values than AL mice (*p* = 0.053) (Figure 2E). No effect was detected for subgroups [LMM, *F*_(1,66)_ = 0.982, η^2^_*p*_ = 0.015, *p* = 0.325) and group x subgroups interaction [LMM, *F*_(2,66)_ = 0.107, η^2^_*p*_ = 0.003, *p* = 0.899].

### 3.2. Only touchscreen training and the feeding regime were found to affect anxiety-like behavior

**Figure 3:**
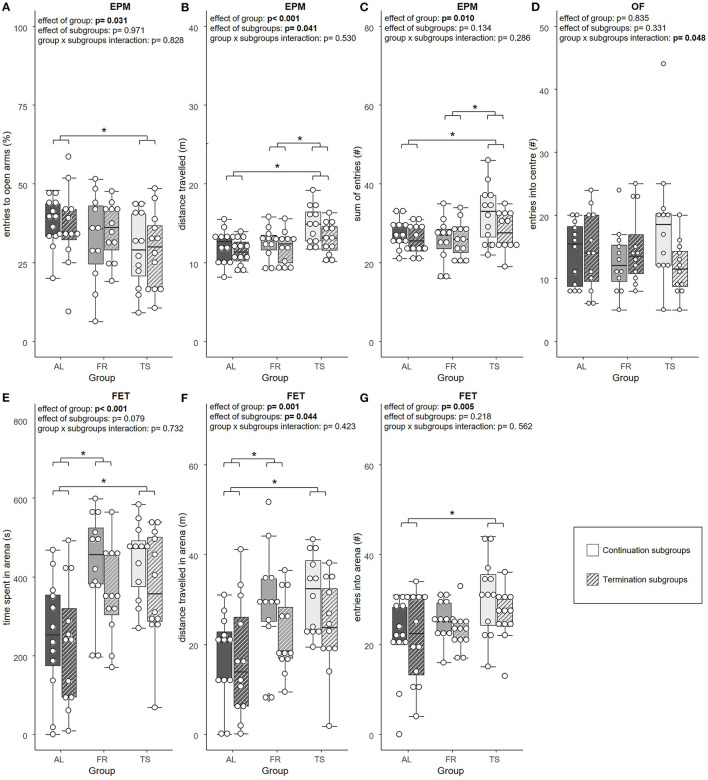
Anxiety-like and exploratory behavior. **(A)** relative number of entries into the open arms of the Elevated plus maze test (EPM), **(B)** total distance traveled in the EPM, **(C)** sum of arm entries in the EPM, **(D)** number of entries into the center of the Open field test (OF), **(E)** time spent in the arena of the Free exploration test (FET), **(F)** total distance traveled in the arena of the FET, **(G)** number of entries into the arena of the FET. AL, *ad libitum* fed mice; FR, food restricted mice; TS, touchscreen trained mice. Sample sizes: *n* = 12/group. Exception: FR mice from continuation subgroups in A, where *n* = 11. Box plots show the median (lines in boxes), the 25 and 75% quartiles (boxes), the minima and maxima (whiskers) and individual data points (circles). Statistics: LMM. *p ≤ 0.05.

In the tests for anxiety-like and exploratory behavior, a significant effect of group was found for the relative number of open arm entries [LMM, *F*_(2,63)_ = 3.658, η^2^_*p*_ = 0.104, *p* = 0.031], distance traveled [LMM, *F*_(2,63)_ = 8.101, η^2^_*p*_ = 0.205, *p* < 0.001] and sum of entries in the EPM [LMM, *F*_(2,63)_ = 4.951, η^2^_*p*_ = 0.136, *p* = 0.010]. Also, the time spent in the arena [LMM, *F*_(2,64)_ = 12.094, η^2^_*p*_ = 0.274, *p* < 0.001], the distance traveled there [LMM, *F*_(2,64)_ = 7.574, η^2^_*p*_ = 0.191, *p* = 0.001] and the number of entries made to the arena of the FET [LMM, *F*_(2,66)_ = 5.684, η^2^_*p*_ = 0.147, *p* = 0.005] were influenced by group. *Post hoc* testing revealed that TS mice made significantly less relative open arm entries compared to AL mice (*p* = 0.025), which suggests increased anxiety-like behavior (Figure 3A). Moreover, TS mice traveled a greater distance than AL (*p* < 0.001) and FR mice (*p* = 0.012) (Figure 3B) and showed more EPM arm entries in total compared to both of the control groups (AL: *p* = 0.046; FR: *p* = 0.013) (Figure 3C), both parameters that indicate increased locomotor behavior. In the FET, TS and FR mice were found to spend more time (TS and FR: *p* < 0.001) (Figure 3E) and travel a greater distance in the FET arena in contrast to AL mice (TS: *p* = 0.002; FR: *p* = 0.012) (Figure 3F). Moreover, TS mice entered the arena more often than AL mice (*p* = 0.004), indicating increased exploratory behavior (Figure 3G). Additionally, there was a trend for an effect of group on the latency to enter the FET arena [LMM, *F*_(2,64)_ = 2.498, η^2^_*p*_ = 0.072, *p* = 0.090]. On a descriptive level, FR and TS mice were faster to enter the arena. No effect of group was detected on relative time spent on the open arms of the EPM and the distance traveled there, as well as on distance traveled in the OF and entries made to and time spent in the center of the OF (LMM, *p* > 0.05 for all comparisons, for details see Supplementary material).

An effect of subgroups was detected for the distance traveled in the EPM [LMM, *F*_(1,63)_ = 4.332, η^2^_*p*_ = 0.064, *p* = 0.041] (Figure 3B) and FET [LMM, *F*_(1,64)_ = 4.203, η^2^_*p*_ = 0.062, *p* = 0.044] (Figure 3F). The continuation subgroups of TS and FR mice traveled a greater distance than the according termination subgroups, indicating increased locomotor behavior. Distance traveled in the OF showed a trend for an effect of subgroups [LMM, *F*_(1,64)_ = 3.971, η^2^_*p*_ = 0.058, *p* = 0.051], with mice from the continuation subgroups traveling slightly more than mice from the termination subgroups. Furthermore, there was a trend for an effect of subgroups on arena time in the FET [LMM, *F*_(1,64)_ = 3.196, η^2^_*p*_ = 0.048, *p* = 0.079], that indicated a tendency for a longer arena time in mice from the continuation subgroups compared to mice from the termination subgroups, which reflect increased exploratory behavior. Subgroups were not found to affect the relative entries made to, the relative time spent on, and the distance traveled on the open arms of the EPM, as well as the sum of arm entries. Also, entries made to the center of the OF, time spent there and latency to enter the arena in the FET and time spent there did not reveal an effect of subgroups (LMM, *p* > 0.05 for all comparisons, for details see Supplementary material).

Only the number of entries made to the center of the OF revealed a significant group × subgroups interaction [LMM, *F*_(2,64)_ = 3.177, η^2^_*p*_ = 0.090, *p* = 0.048]. However, *post hoc* testing did not detect any significant differences (Figure 3D). None of the other parameters from the EPM, OF and FET showed a group × subgroups interaction effect (LMM, *p* > 0.05 for all comparisons, for details see Supplementary material).

### 3.3. The feeding regime altered home cage behavior

**Figure 4:**
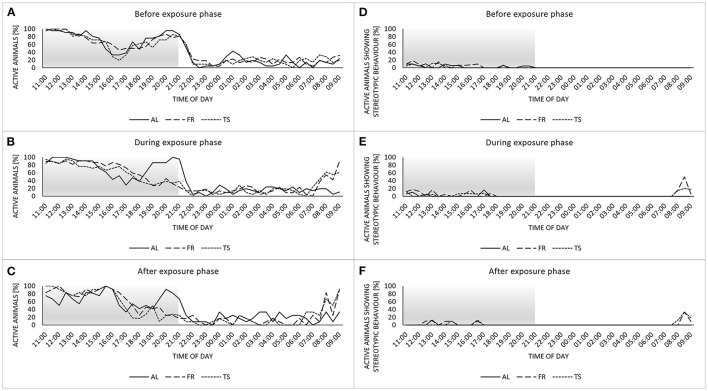
Home cage behavior. **(A–C)** percentage of active individuals per sample point divided by group (AL, *ad libitum* fed mice; FR, food restricted mice; TS, touchscreen trained mice) for the recording times before **(A)**, during **(B)** and after **(C)** the exposure phase. **(D–F)** percentage of active animals showing stereotypic behavior divided by group for the recording times before **(D)**, during **(E)** and after **(F)** the exposure phase. Sample sizes: *n* = 24/group for **(A–E)** and *n* = 12/group for **(C, F)**. Sample sizes can vary slightly between groups and sample points, due to technical issues with the camera system (for details see Supplementary material). Gray area highlights the dark phase.

Concerning the home cage behavior, mice showed a biphasic activity rhythm before the exposure phase, with two activity peaks divided by a rest, and no noticeable differences between the three groups (Figure 4A). During the exposure phase, the activity rhythms of TS and FR mice changed into a more monophasic profile, with a steady decrease in activity from morning to night (Figure 4B). Also, the onset of activity began earlier compared to AL mice. These differences were maintained after the termination of touchscreen training for TS mice from the termination subgroups (Figure 4C). The display of stereotypic behavior was very low in general and mainly restricted to the dark and therefore active phase of the animals (Figures 4D–F). However, during the exposure phase and beyond, TS and FR mice showed a peak in stereotypic behavior between 8 a.m. and 9 a.m. that could not be observed in AL mice.

## 4. Discussion

The aim of the present study was to shed more light on recently reported touchscreen training effects, with a particular focus on the termination of the training routine. Two main patterns emerged: First, we confirmed previous findings showing that a restricted feeding regime as an integral part of touchscreen training affects the animals' behavior and activity. Secondly, touchscreen training increased FCMs and anxiety-like behavior of the mice. With regard to our main hypothesis, however, no effect of the termination of touchscreen training could be detected.

### 4.1. The feeding regime affects exploratory behavior and home cage activity

In the behavior tests as well as in the animals' home cage behavior, effects of the mild food restriction were detected. Regarding the behavior tests, both TS and FR mice showed increased levels of exploratory behavior compared to *ad libitum*-fed animals. This is in line with previous findings showing that a restrictive diet can increase exploration (e.g., Day et al., 1995) and likely reflects a higher motivation of the animals to forage for food. Moreover, TS and FR mice displayed differences in home cage behavior compared to the *ad libitum* fed group. Stereotypic behavior, an indicator of impaired welfare, was slightly increased around the time of exposure to the respective experimental procedures. Yet, the absolute values were too low to allow final conclusions. A comparable increase in stereotypic behavior due to a restricted diet was already reported before when investigating the effects of different food restriction routines (Feige-Diller et al., 2020). Overlapping with the small peak in stereotypies was a peak in activity, also shown by both TS and FR mice. Such an activity-related adaptation to a certain feeding routine, also known as food entrainment, is assumed to reflect anticipatory arousal (Krieger, 1974; Stephan, 2002; Gooley et al., 2006; Refinetti, 2015; Feige-Diller et al., 2020). Activity levels of TS and FR mice not only differed from AL mice shortly before the daily feeding event, but were also shifted during the course of the day. While AL mice displayed a biphasic activity rhythm, which was also reported before in C57BL/6J mice (Bodden et al., 2019), TS and FR mice changed their activity with the onset of the new diet into a more monophasic rhythm. Yet, a welfare-related evaluation of this shift in activity compared to AL mice would be inconclusive, as an *ad libitum* diet has been severely criticized as an appropriate feeding regime for laboratory rodents (for a review see Keenan et al., 1996). Taken together, the observed changes in behavior and activity caused by restricted feeding, which is an integral part of touchscreen training, have important implications for future experiments, as different activity states can affect the performance in other behavior tests as well as the reproducibility of results (Bodden et al., 2019).

### 4.2. Touchscreen training affected FCMs and anxiety-like behavior

The second main result was that touchscreen training affects HPA axis activity and anxiety-like behavior. Regarding the FCM analysis, touchscreen trained mice showed elevated FCM reaction values. This is consistent with our previous study (Krakenberg et al., 2021), as well as with the results of Mallien et al. (2016), who detected an increase of serum corticosterone in direct anticipation of a training session. Notably, the time directly before training is also reflected in the reaction values we measured. Thus, our results confirm a state of increased arousal in anticipation of and during touchscreen training. As in our previous study, FCM reaction values were still increased after the termination of training, indicating that the anticipation of training persists even beyond the training phase itself (Krakenberg et al., 2021). In contrast to the reaction values, baseline FCMs were not found to differ between the groups. This is also in line with the literature, where a decrease of FCMs back to baseline ~2 h after the training sessions has been reported (Mallien et al., 2016; Krakenberg et al., 2021). Thus, the animals' state of increased arousal can be assumed to be rather transient, peaking around the time of exposure and decreasing again shortly afterwards.

At first glance, these results might point toward a putatively negative impact of touchscreen training on the welfare of mice, as, traditionally, elevated corticosterone levels are associated with aversive situations [e.g., predator confrontation (Amaral et al., 2010)]. Yet, increased adrenocortical activity can also be observed in reaction to beneficial stimuli [e.g. environmental enrichment (Marashi et al., 2003), see also Koolhaas et al., 2011 for a review]. Particularly the decrease of FCMs back to baseline levels indicates successful coping and the absence of chronic stress caused by the regular training sessions. Thus, the observed hormonal effects could also be interpreted in terms of a potentially enriching effect of touchscreen training by reducing under-stimulation many laboratory animals face (Wemelsfelder, 1985; van Rooijen, 1991; Burn, 2017; Meagher, 2019).

However, in addition to these effects on HPA axis activity, TS mice showed increased levels of anxiety-like behavior, an overall effect that was not dependent on whether TS mice were still trained at the point of testing or not. Specifically, this was reflected in the relative number of open arm entries in the EPM. In our previous study, also other parameters reflecting anxiety-like behavior (e.g., relative open arm time in the EPM) differed significantly between touchscreen-trained and control mice but we can only speculate about the reasons for this. However, descriptively, the present data point into the same direction. This is further underlined by another study conducted at our lab, although with a different research focus: Bračić et al. (2022) also detected increased anxiety-like behavior in touchscreen-trained mice. Again, the respective parameters reflecting anxiety-like behavior differed partly from the two above mentioned studies (e.g., time in the center of the OF). Taken together, there is mounting evidence for touchscreen training to increase anxiety-like behavior in mice, even though the specific parameters reflecting this effect may vary. Moreover, since Bračić et al. investigated female mice, including animals of both the C57BL/6J and B6D2F1 strain, the effect might even be robust across sexes and strains, however, caution is still advisable when generalizing these results.

At the same time, the findings of this study demonstrate that the termination of training is not the critical factor triggering the observed increase in anxiety-like behavior in touchscreen-trained animals. Therefore, the “enrichment loss hypothesis” could not be confirmed in the present study.

Traditionally, increased anxiety-like behavior, similarly to increased FCMs, would be interpreted as an indicator of a negative affective state (Paul et al., 2005; Hurst and West, 2010), suggesting a putatively negative impact of training on our touchscreen groups. As previously argued, however, one alternative explanation for the increased anxiety-like behavior might exist: a potential “negative contrast effect” (Krakenberg et al., 2021). Briefly, a negative contrast emerges if an individual anticipates a rewarding event, but a comparably less rewarding event actually occurs (e.g., Flaherty, 1982). If touchscreen training was indeed perceived as enriching by the mice, their training anticipation might have been disappointed by being placed on the tests for anxiety-like behavior and not into the touchscreen chamber. This might have caused a negative affective state, reflected in their anxiety-like behavior (Krakenberg et al., 2021).

Taken together, the current state of knowledge is not sufficient to draw final conclusions at this stage, which is why further studies on the effects of touchscreen training are necessary. Yet, the present study successfully reproduced previous findings, showing that (I) a mild food restriction increases exploratory behavior and is capable of shifting the activity rhythm of mice, and (II) that regular touchscreen training transiently increases HPA axis activity and leads to higher levels of anxiety-like behavior. Furthermore, this study provides first evidence that these effects are not caused by the termination of regular touchscreen training. In compliance with the refinement endeavors for laboratory animals, further research should aim for a thorough assessment of the procedure's severity to ensure a responsible and well-founded use of animals for experimental purposes.

## Data availability statement

The raw data supporting the conclusions of this article will be made available by the authors upon request.

## Ethics statement

All procedures complied with the regulations covering animal experimentation within Germany (Animal Welfare Act) and the EU (European Communities Council DIRECTIVE 2010/63/EU) and were approved by the local (Gesundheits- und Veterinäramt Münster, Nordrhein-Westfalen) and federal authorities (Landesamt für Natur, Umwelt und Verbraucherschutz Nordrhein-Westfalen “LANUV NRW”, reference number: 81-02.04.2020.A120).

## Author contributions

SHR and VS conceived the study and supervised the project. SK, SHR, NS, and VS designed the experiments. VS trained SQ in conducting the experiments. SQ carried out the experiments. RP determined and analyzed the hormonal data. SQ and VS conducted the statistical analysis of the data and wrote the initial draft of the manuscript. All authors critically revised the manuscript and gave final approval for publication.
