# Supplementary material for: The power of a touch: Regular touchscreen training but not its termination affects hormones and behavior in mice
Source: Front Behav Neurosci. 2023 Mar 16;17:1112780. doi: 10.3389/fnbeh.2023.1112780 (PMC10060536; doi:10.3389/fnbeh.2023.1112780)
Supplement: Supplementary file 1 [file Data_Sheet_1.PDF]

Supplementary Material

Touchscreen (TS) training:

Setup:

The TS setup consisted of four Bussey-Saksida mouse TS chambers (Bussey-Saksida Mouse Touch Screen Chamber Package, Model 80614, Campden Instruments LTD, Loughborough, Leics., United Kingdom) that were located in isolation chambers in an easy-install system (Easy-Install System for Mouse Touch Screen Systems, Model 80614-20, Campden Instruments LTD, Loughborough, Leics., United Kingdom). All chambers were connected to a computer (Controller PC – Touch Screen Chambers, Model 88530, Campden Instruments LTD, Loughborough, Leics., United Kingdom) and were equipped with cameras to film the inside under infrared lighting conditions (Camera, filtered and focussed for infrared light, Model 80600-CAM, Campden Instruments LTD, Loughborough, Leics., United Kingdom). The cameras were connected to a video recorder (Digital Video Recorder WBXRa040E, WBox Technologies, Analog Devices Inc. Global Distribution, Norwood, United States). The videos could be accessed via a digital interface (Model 81426, Campden Instruments LTD, Loughborough, Leics., United Kingdom). The operating system used was WhiskerServer (Version 4.5.0, Cambridge University Technical Services Ltd, Cambridge, United Kingdom) and the software was Abet II Touch (Version 2.20, Model 89505, Lafayette Instruments Company, Lafayette, United States).

Training programme:

The exemplary TS training programme used within this study was originally designed to prepare mice for a cognitive judgement bias task (e.g. Krakenberg et al., 2019a) and was only adjusted slightly. The maximum time for each step of training was set to 15 minutes and there was no trial limit in any of the steps. After the 8th training step (see below), mice did not proceed to the next step, as the final goal of the study was not to test the mice in the cognitive judgment bias test (for an overview of training steps see Tab. 1). However, the visual cues used were the same as in the original programme (Fig. 1). These cues were presented to the mice on a black background within the three cutouts through which the TS was accessible. In the centre cutout, a white, horizontal bar (height: 1.1 cm, length: 6.3 cm) that could vary in vertical position was presented. This field was never touch sensitive. In the left and right cutouts, crosses were displayed that were formed by two intersecting bars (height: 1.1 cm, length: 6.3 cm). These fields were both touch sensitive.

In the 1st step, the mice had one day to familiarise with the TS chamber and the reward, which was sweet condensed milk (Milchmädchen, Nestlé, Frankfurt am Main, Germany), diluted 1:4 in tap water. A reward was delivered together with the onset of the tray light and a tone (frequency: 3 kHz, duration: 1000 ms). The initial reward had a size of 150 µl to motivate the animals for reward collection. The following reward deliveries had a size of 7 µl. Between all trials there was an intertrial interval of 10 seconds and new trials started after reward collection only. In this step, the mice were supposed to learn where to get a reward and to associate it with the onset of the tray light and the tone.

In the 2nd step, a cross was displayed on either the left or the right TS field in each trial in a balanced way. If the mouse touched the cross, a reward of 21 µl was delivered. If not, the cross disappeared after

30 seconds and a reward of 7 µl was delivered. The intertrial interval in this and further steps was 5 seconds after reward collection. To proceed to the next step, the mice had to reach 15 trials in 15 minutes.

After successful completion, the animals were trained in the 3rd step. Here, the setting was like the one in the 2nd step, except that a successful touch was rewarded with 7 µl of the reward, while a missed touch was unrewarded. The criterion to enter the next step was 15 trials in 15 minutes on two consecutive training days.

In the following 4th step, the mice had to actively initiate each trial by entering the tray that was illuminated before the start of each trial. A successful initiation was indicated by a click sound (duration: 200 ms). To trigger the first initiation, the illumination of the tray was accompanied by a reward delivery of 7 µl and the tone. From this point on, active trial initiation was needed for all following steps. The remaining settings were as in the previous step. Animals that reached 15 trials in 15 minutes progressed to the next step.

After the mice learned to initiate trials, they were trained in the 5th step. The only difference to the past step was that the mice could also touch incorrectly by touching the side that did not show the cross. Whenever this happened, the cross disappeared and the trial was counted as incorrect. The mice received a mild punishment, consisting of the illumination of the house light inside the TS chamber for five seconds, accompanied by a time out of 5 seconds, in which no new trial could be initiated. This mild punishment remained the same during the whole training programme. After each incorrect trial, correction trials were presented, in which the incorrectly completed trial was repeated until the mouse did it correctly. To finish this step, the mice had to do 15 trials in 15 minutes with a correctness of 85 % on two consecutive training days.

The 6th step was the first in which a bar was presented as a cue in the central field of the TS. This bar was either at the bottom or at the top of the field. Again, the appearance of the bottom or top bar was balanced across trials and a bar was never shown in the same place more than three times in a row. With a delay of 2 seconds, crosses were presented in the left and right TS field as well. To avoid side effects, the correct touch side was always counterbalanced. However, during the 6th step, the wrong cross (small reward in the positive condition and mild punishment in the negative condition) was blocked. Correct touches were rewarded with 7 µl of the reward. To finish this step, the mice had to do 25 trials in 15 minutes on two consecutive training days.

The following 7th step started with 10 trials of the 6th step, where only the correct cross was touch sensitive. After these ten trials, the crosses appeared at the same time as the bar and both crosses were touch sensitive. When touching correctly in the positive condition, the mice received a big reward of 12 µl and a tone sounded for 1000 ms. In the positive and the negative condition, the small reward had a size of 4 µl and was accompanied by a tone with the duration of 333 ms. The mild punishment in the negative condition was as described earlier and again, correction trials were presented when a mouse touched incorrectly. To reach the next step, the mice had to respond to the negative and positive cue with a correctness of 80 % each on two consecutive training days. Furthermore, the number of correction trials was not allowed to exceed 20, except on the first day in this step. If animals did more than 20 correction trials after their first day of training in the present step, they were returned to the 6th step of training.

For the 8th and last step of training the settings were as in the preceding step, but this time only 4 trials of the 6th training step, where only the correct cross was touch sensitive, were presented at the beginning. In this step, the threshold to be returned to the 6th step of training was 30 correction trials.

By the end of the study, 6 of 12 mice from the TS continuation subgroup (7 weeks of training) were in the 7th step, while the remaining 6 mice were in the 8th step. For the TS termination subgroup (5 weeks of training), 10 of 12 mice were in the 7th step and 2 mice were in 8th step.

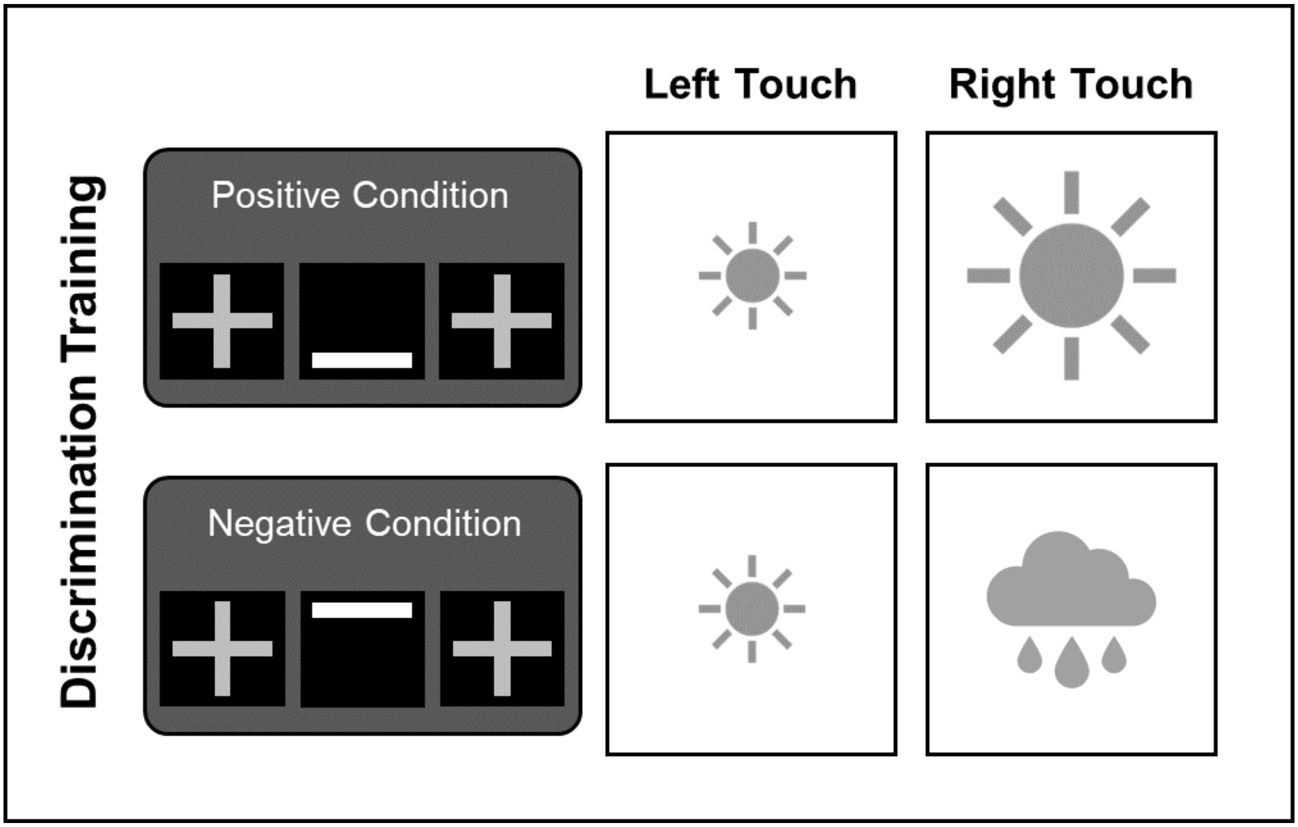

Supplementary Figure 1. Schematic overview of the discrimination task in the touchscreen training. Depicted is a schematic illustration of the discrimination task in touchscreen training, in which a small (small sun) or a large reward (large sun) can be obtained in the positive condition (bar in bottom position), while in the negative condition (bar in top position) a small reward (small sun) or a mild punishment (rain cloud) can be obtained. Figure adopted from Krakenberg et al. (2019a) with permission from Elsevier.

Supplementary Table 1. Overview of touchscreen training steps. Shown are the different steps during touchscreen training, their duration, trial limit and the criteria to proceed to next step, respectively to return to a previous step.

Step	Max. time (min)	Task	Criterion to proceed to next step	Criterion to return to a respective training step
1	15	Habituate to touchscreen chamber	-	-
2	15	Touch the cross (bigger reward for correct touch, smaller reward for missed touch, field without cross is blocked)	15 trials in 15 min on 1 day	lack of motivation if less than 5 trials on 10 days<br>➔ **exclude**
3	15	Touch the cross (smaller reward for correct touch, no reward for missed touch, field without cross is blocked)	15 trials in 15 min on 2 consecutive training days	-
4	15	Initiate trials	15 trials in 15 min on 1 day	-
5	15	Touch the cross (correct touch is rewarded, incorrect touch is mildly punished)	15 trials in 15 min on 2 consecutive training days, each with 85% correct (13/15)	if less than 8 trials on 5 consecutive days or less than 12 trails on 10 days<br>➔ **return to step 2**
6	15	Learn cues (middle bar is presented, touch of the correct cross is rewarded, incorrect cross is blocked)	25 trials on 2 consecutive training days	lack of motivation if less than 10 trials on<br>15 days<br>➔ **return to step 3**
7	15	Learn cues (after 10 trials of step 6 both crosses are touch sensitive)	10 trials in 15 min on 2 consecutive days, each with 80% correct (8/10) positive & negative cue	if the number of correction trials, except on 1. training day, **exceeds 20**<br>➔ **return to step 6**<br>the precondition to stay, was that on the next training days, the number of correction trials had to be reduced by at least 45 % daily.

				Alternatively, the animals also stayed, when the number of correction trials reached 20 or less on the next training day
8	15	Learn cues (after 4 trials of step 6 both crosses are touch sensitive)	25 trials in 15 min on 2 consecutive days, each with 80% correct (20/25) positive & negative cue	if the number of correction trials, except on 1. training day, **exceeds 30**<br>➔ **return to step 6**<br>the precondition to stay, was that on the next training days, the number of correction trials had to be reduced by at least 45 % daily. Alternatively, the animals also stayed, when the number of correction trials reached 30 or less on the next training day

Food restriction:

With the start of the exposure phase, mice from the FR group and the TS group were mildly food restricted in a way that they maintained 90-95 % of their highest weight measured during the handling week or the first day of TS training. Mice from the AL group still received food *ad libitum*. To avoid large bodyweight fluctuations, each mouse received an individually adjusted amount of food, based on its current percentage bodyweight that was calculated by daily weighing (PCE-BT 2000, PCE Deutschland GmbH, Meschede, Germany; weighing capacity: 2100 g, resolution: 0.01 g). Therefore, food pellets of different weights (2.3-4.9 g) were used. These pellets were cut precisely to one decimal place after the comma from standard housing food. The feeding was integrated into the experimental procedure and occurred between 9 a.m. and 11 a.m.

frontiers

Statistical analysis of behavioural test parameters:

Supplementary Table 2. Behavioural test parameters. Data are presented as untransformed means for the three groups (TS= touchscreen trained, FR= food restricted, AL= ad libitum fed), the two subgroups (continuation and termination) and the groups divided by subgroup (TS, FR, AL from continuation subgroups, respectively termination subgroups) ± SD. Sample sizes: n= 12/ group. Exception: FR mice from continuation subgroups in EPM, where n= 11.

	Mean±SD										
						Group*Subgroups					
	Group			Subgroups		continuation			termination		
	TS	FR	AL	continuation	termination	TS	FR	AL	TS	FR	AL
Elevated plus maze test											
Time spent on open arms (%)	21.5 ±11.3	25.2 ±10.8	26.1 ±8.4	26.1 ±10.6	22.5 ±9.9	23.2 ±11.7	26.4 ±12.4	28.6 ±6.2	19.9 ±10.7	24.2 ±9.0	23.6 ±9.5
Entries into open arms (%)	28.9 ±11.5	33.8 ±11.8	37.1 ±10.1	33.1 ±11.7	33.4 ±11.3	28.9 ±11.4	32.5 ±13.5	38.0 ±7.6	28.9 ±11.7	35.0 ±8.6	36.2 ±12.0
Distance travelled on open arms (m)	1.8 ±1.2	1.9 ±1.0	2.1 ±1.0	2.1 ±1.0	1.7 ±1.1	2.0 ±1.1	2.1 ±1.1	2.3 ±0.8	1.6 ±1.2	1.8 ±0.9	1.8 ±1.1
Distance travelled (m)	14.0 ±2.3	12.2 ±1.9	11.7 ±1.9	13.2 ±2.4	12.1 ±2.0	14.9 ±2.3	12.5 ±1.9	12.1 ±2.1	13.1 ±1.9	11.9 ±1.9	11.4 ±1.6

Sum of entries (#)	30.3 ±6.3	25.9 ±4.9	26.7 ±3.6	28.6 ±6.2	26.7 ±4.3	32.4 ±7.0	25.7 ±5.4	27.4 ±3.6	28.1 ±4.7	26.1 ±4.3	26.0 ±3.6
Open field test											
Time spent in centre (s)	17.9 ±8.3	18.0 ±8.3	16.6 ±7.8	17.3 ±8.7	17.7 ±7.6	19.5 ±10.6	15.7 ±6.2	16.7 ±8.2	16.3 ±4.4	20.4 ±9.4	16.5 ±7.4
Entries into centre (#)	15.1 ±7.9	13.7 ±5.3	14.2 ±5.4	15.1 ±7.1	13.6 ±5.4	18.5 ±9.3	12.7 ±4.8	14.1 ±4.7	11.7 ±4.0	14.8 ±5.5	14.3 ±6.0
Distance travelled (m)	41.4 ±9.2	37.2 ±5.7	39.4 ±7.2	41.1 ±8.8	37.5 ±6.0	45.4 ±10.6	38.1 ±6.4	39.9 ±6.9	37.4 ±5.1	36.4 ±4.8	38.9 ±7.4
Free exploration test											
Distance travelled in arena (m)	27.6 ±9.9	25.7 ±10.8	16.8 ±11.2	25.8 ±11.8	20.9 ±10.9	31.3 ±8.3	29.1 ±11.9	17.1 ±9.5	23.9 ±9.9	22.2 ±8.3	16.6 ±12.6
Latency to enter arena (s)	36.5 ±42.0	36.9 ±41.9	84.8 ±174.6	61.4 ±146.1	44.1 ±46.8	28.3 ±30.7	30.3 ±33.3	125.6 ±236.2	44.6 ±49.5	43.6 ±48.2	43.9 ±42.4
Time spent in arena (s)	407.7 ±122.0	395.3 ±124.4	239.5 ±147.1	375.3 ±151.3	319.7 ±148.1	442.7 ±92.1	433.3 ±126.9	250.0 ±143.2	372.7 ±137.3	357.3 ±109.5	229.1 ±150.2
Entries into arena (#)	28.7 ±7.4	24.2 ±4.3	21.5 ±9.2	25.9 ±8.3	23.7 ±7.1	30.8 ±8.3	25.4 ±4.2	21.4 ±8.6	26.5 ±5.5	23.0 ±4.1	21.7 ±9.7

Supplementary Table 3. Statistical analysis of behavioural test parameters. Statistical information given: main effect of group, subgroups and group*subgroups (LMM: F-ratio, p-value), transformation used (SQRT= square root transformation, log10= logarithmic transformation). Sample sizes: n= 12/ group. Exception: FR mice from continuation subgroups in EPM, where n= 11. Bold: p-values indicating a significant effect.

	LMM									
	**Group**			**Subgroups**			**Group*Subgroups**			
	**F**	**η^2_p**	**p**	**F**	**η^2_p**	**p**	**F**	**η^2_p**	**p**	**Transfor-mation**
**Elevated plus maze test**										
Time spent on open arms (%)	1.589	0.048	0.212	2.608	0.040	0.111	0.092	0.003	0.912	-
Entries into open arms (%)	3.658	0.104	**0.031**	0.001	<0.001	0.971	0.190	0.057	0.828	-
Distance travelled on open arms (m)	0.827	0.026	0.442	2.199	0.033	0.143	0.091	0.003	0.913	SQRT
Distance travelled (m)	8.101	0.205	**<0.001**	4.332	0.064	**0.041**	0.641	0.020	0.530	-
Sum of entries (#)	4.951	0.136	**0.010**	2.299	0.035	0.134	1.276	0.039	0.286	-
**Open field test**										
Time spent in centre (s)	0.335	0.010	0.717	0.189	0.003	0.665	0.833	0.025	0.439	log10
Entries into centre (#)	0.181	0.006	0.835	0.961	0.015	0.331	3.177	0.090	**0.048**	SQRT
Distance travelled (m)	1.773	0.052	0.178	3.971	0.058	0.051	1.476	0.044	0.236	SQRT
**Free exploration test**										
Distance travelled in arena (m)	7.574	0.191	**0.001**	4.203	0.061	**0.044**	0.871	0.026	0.423	-

Latency to enter arena (s)	2.498	0.072	0.090	0.007	<0.001	0.934	1.613	0.048	0.207	log10
Time spent in arena (s)	12.094	0.274	**<0.001**	3.196	0.048	0.079	0.314	0.010	0.732	-
Entries into arena (#)	5.684	0.147	**0.005**	1.544	0.023	0.218	0.581	0.017	0.562	-

Sample sizes for the home cage behaviour recordings:

Supplementary Table 4. Sample sizes for the home cage behaviour recordings. Shown are the sample sizes from the home cage behaviour recordings for each group at each sample point during each phase of the experiment.

	Before exposure phase			*During exposure phase*			*After exposure phase*		
Sample point	AL	FR	TS	AL	FR	TS	AL	FR	TS
11:00	21	22	21	19	19	18	12	12	12
11:30	21	22	21	19	19	18	12	12	12
12:00	21	22	21	19	19	18	12	12	12
12:30	21	22	21	21	22	21	12	12	12
13:00	21	22	21	21	22	21	12	12	12
13:30	21	22	21	21	22	21	11	11	12
14:00	21	22	21	21	22	21	12	12	12
14:30	21	22	21	21	22	21	11	12	11
15:00	21	22	21	21	22	21	12	12	12
15:30	21	22	21	21	22	21	12	11	11
16:00	21	22	21	21	22	21	12	12	12
16:30	21	22	21	21	22	21	12	12	12

17:00	21	22	21	21	22	21	12	12	12
17:30	21	22	21	21	22	21	11	12	11
18:00	21	22	21	21	22	21	12	12	12
18:30	21	22	21	21	22	21	10	11	11
19:00	21	22	21	21	22	21	12	12	12
19:30	21	22	21	21	22	21	12	11	11
20:00	21	22	21	21	22	21	12	12	12
20:30	21	22	21	21	22	21	11	12	11
21:00	21	22	21	21	22	21	12	12	12
21:30	21	22	21	21	22	21	12	12	12
22:00	21	22	21	21	22	21	12	12	12
22:30	21	22	21	21	22	21	12	12	12
23:00	21	22	21	21	22	21	12	12	12
23:30	21	22	21	21	22	21	12	11	11
00:00	21	22	21	21	22	21	12	12	12
00:30	21	22	21	21	22	21	12	12	12
01:00	21	22	21	21	22	21	12	12	12
01:30	21	22	21	21	22	21	12	12	12
02:00	21	22	21	21	22	21	12	12	12
02:30	21	22	21	21	22	21	12	12	12
03:00	21	22	21	21	22	21	12	12	12
03:30	21	22	21	21	22	21	12	12	12

04:00	21	22	21	21	22	21	12	12	12
04:30	21	22	21	21	22	21	12	12	12
05:00	21	22	21	21	22	21	12	12	12
05:30	21	22	21	21	22	21	12	12	12
06:00	21	22	21	21	22	21	12	12	12
06:30	21	22	21	21	22	21	12	12	12
07:00	21	22	21	21	22	21	12	12	12
07:30	21	22	21	21	22	21	12	12	12
08:00	21	22	21	21	22	21	12	12	12
08:30	21	22	21	19	19	18	12	12	12
09:00	21	22	21	19	19	18	12	12	12

References:

Krakenberg, V., Woigk, I., Garcia Rodriguez, L., Kästner, N., Kaiser, S., Sachser, N., & Richter, S. H. (2019a). Technology or ecology? New tools to assess cognitive judgement bias in mice. *Behavioural Brain Research*, *362*(December 2018), 279–287.
